# Up-to-Date Snapshot of Current and Emerging Medical Therapies in Primary Biliary Cholangitis

**DOI:** 10.3390/jpm14121133

**Published:** 2024-11-30

**Authors:** Zakary Warsop, Nikhil Anand, Husam Al Maliki, Shuell De Souza, Arya Kamyab, Amin Al Hadad, Laith Alrubaiy

**Affiliations:** 1 Gastroenterology and Hepatology Department, Imperial College London, London SW7 2AZ, UK; zakary.warsop18@imperial.ac.uk (Z.W.); nikhil.anand18@imperial.ac.uk (N.A.); husam.al-maliki21@imperial.ac.uk (H.A.M.); shuell.de-souza18@imperial.ac.uk (S.D.S.); arya.kamyab21@imperial.ac.uk (A.K.); 2 Healthpoint Hospital, Abu Dhabi 112308, United Arab Emirates; m.alhadad@healthpoint.ae; 3 Department of Medicine Health and Life Sciences, Singleton Bay Campus, Swansea University School of Medicine, Swansea SA2 8PP, UK

**Keywords:** primary biliary cholangitis, primary biliary cirrhosis, PBC, ursodeoxycholic acid, obeticholic acid, bezafibrate, fenofibrate

## Abstract

**Background/Objectives:** Primary biliary cholangitis (PBC) is an autoimmune chronic cholestatic disease of the liver that symptomatically can present with pruritus and fatigue. Its established first- and second-line therapies are ursodeoxycholic acid (UDCA) and obeticholic acid (OCA) although they provide limited symptom management. Liver transplantation offers a potentially curative therapeutic option in refractory cases progressing to cirrhosis. Novel research published after the current guidelines highlights the importance of providing an up-to-date analysis of treatment options available. **Methods:** In this study, we conducted a literature search using Pubmed, Ovid Medline, and SCOPUS to provide a narrative review of first-line, second-line, and emerging therapies in PBC. **Results:** UDCA has been well established as a long-term, safe therapy within the literature although it is possible that treatment dosage can be further optimised in refractory patients. It has a favourable side effect profile. Despite improving biochemical markers, histopathological profile, and overall outcomes, up to 30–40% of patients are refractory to it. Age and sex are highlighted as independent indicators of non-responsiveness. This necessitates effective second-line therapies. Future trials could aim to investigate UDCA as a co-first-line therapy. Further supporting results for OCA were found in the interim extension trial of the seminal POISE study. The long-term phase 4 COBOLT trial is still awaiting results to further assess the complications, adherence, and potential adverse effects. It is a viable option in UDCA-refractory patients. The high incidence rate of dose-related pruritis indicates that alternative second-line options are needed. Bezafibrate is an off-label antilipemic agent that shows promise as a prospective second-line therapy option. The landmark BEZURSO trial alleviated some efficacy and safety concerns, but it remains associated with elevated serum creatinine; thus, it should be considered with caution. Other prospective second-line therapies are budesonide, triple therapy, and novel agents such as seladelpar and elafibranor. **Conclusions:** UDCA should remain the treatment of choice for PBC, though perhaps not as monotherapy. With further investigation, BF shows promise as a new second-line therapy alongside OCA, which it may outperform.

## 1. Introduction

Primary biliary cholangitis (PBC) is an autoimmune chronic cholestatic liver disease, with a prevalence of approximately 20–35/100,000 in the UK and is ten times more common in middle-aged women [1,2,3,4,5,6,7].

The aetiology of PBC is multifactorial and includes genetic predisposition and environmental factors [8]. PBC has a high 63% concordance rate in monozygotic twins and an increased prevalence of other autoimmune diseases such as scleroderma is seen in these patients, indicating a genetic predisposition [9,10]. Cigarette smoking and recurrent urinary tract infections are examples of environmental risk factors for PBC [11,12,13].

The pathogenesis of PBC remains unclear. It is postulated that the loss of immune T-cell self-tolerance against mitochondrial antigens causes the production of antimitochondrial antibodies (AMAs), which are found in 85% of patients with PBC. AMAs bind to lipoic acid containing the E2 component of the pyruvate dehydrogenase complex that is located on the mitochondrial inner membrane. This immune reaction leads to the gradual destruction of intrahepatic bile ducts, resulting in periportal inflammation [1,8,14]. The consequent cholestasis stimulates a proinflammatory response, leading to liver fibrosis, cirrhosis, and potentially liver failure [1,8,14].

Patients with PBC can be asymptomatic and are found incidentally during the evaluation of abnormal liver blood tests. Symptomatic patients can present with jaundice, pruritus, and fatigue [15,16,17]. As the disease progresses, liver decompensation leads to jaundice, ascites, and ultimately cirrhosis [15,16,17,18]. Malabsorption and steatorrhea have also been reported [16,17,18]. Other complications include hyperlipidaemia and osteoporosis, which occurs in 20–37% of cases, often necessitating a risk assessment [15,17,19,20].

**Figure 1:**
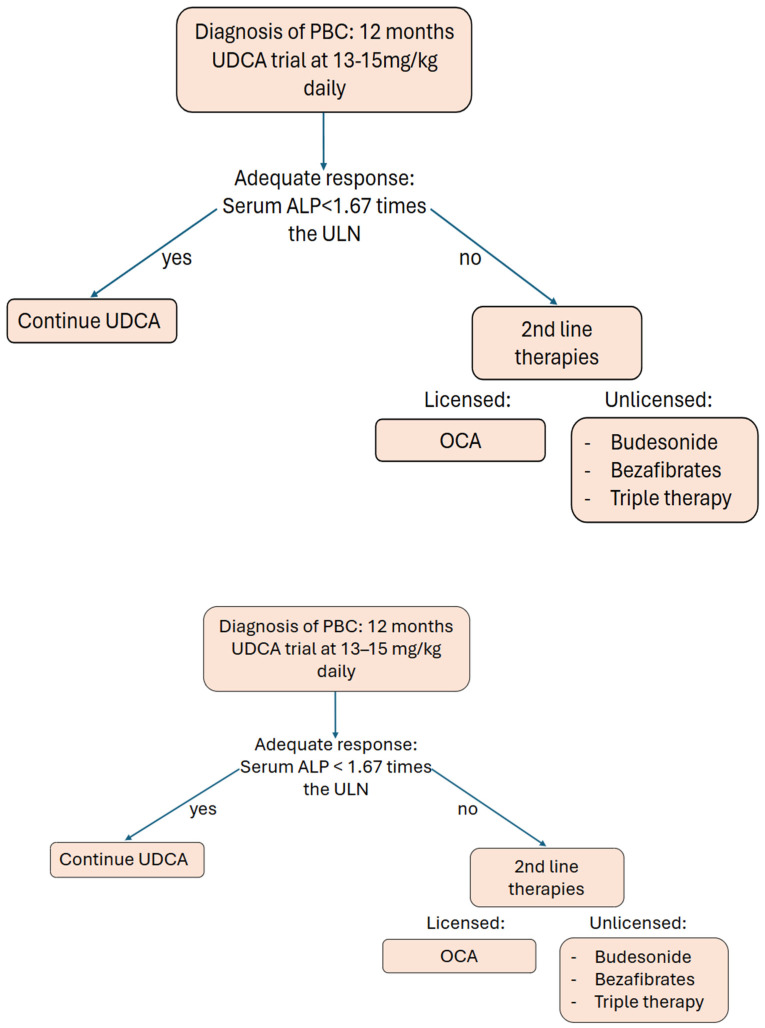
Pathway for patient care in regard to disease-modifying management of PBC. PBC, Primary Biliary Cholangitis; UDCA ursodeoxycholic acid; ALP, alkaline phosphatase; ULN, upper limit of normal; OCA, Obetacholic Acid.

Following the exclusion of other liver diseases, the diagnosis of PBC is made if two of the three following criteria are met, i.e., (1) presence of cholestatic liver biochemistry (significant elevation in alkaline phosphatase (ALP) and gamma glutamyl transferase (GGT) levels), (2) presence of specific PBC autoantibodies, (3) and histopathological characteristics of PBC (Figure 1) [15,16,17,21]. A liver biopsy is unnecessary where coexisting liver disease is not suspected [15,16,17,22].

The therapy of primary biliary cholangitis aims to prevent disease progression, manage symptoms, and prevent complications related to chronic cholestasis [23,24,25,26,27,28,29,30,31]. Response to treatment can be defined by several criteria, and when compared to each other, there is no significant difference between the criteria (Table 1) [32]. The aim of this study is to provide an up-to-date literature review of the effectiveness of first- and second-line treatments as well as emerging novel therapies for PBC. In order to do this, we searched Pubmed, Ovid Medline, and Web of Science databases from 10 January 2022 for articles investigating medical therapy for PBC (Table 2). Randomised controlled trials (RCTs) and other primary articles were prioritised.

## 2. Medical Therapies for Primary Biliary Cholangitis (PBC)

The available and emerging medical therapies for PBC are summarised in Table 3.

The above therapies are mentioned in guidelines across Europe, European Association for the study of the Liver (EASL); America, American College of Gastroenterology (ACG); and the UK, The British Society of Gastroenterology (BSG) (Table 4).

EASL, BSG, and ACG treatment guidelines focus on reduction in mortality and liver transplantation.

EASL as well as BSG recommend that the management of PBC should involve the use of UDCA in all patients, and then, the patient’s biochemical response should be reassessed after 1 year to identify high-risk patients [15,16], where inadequate response indicates using OCA as a dual therapy. These are patients that have an ALP > 1.67× ULN and bilirubin < 2× ULN. OCA is also used in monotherapy in patients that cannot tolerate UDCA. Alternate therapies are not currently licensed, and as such, EASL and BSG maintain that a recommendation regarding budesonide and bezafibrate cannot be made.

ACG and the chronic liver disease foundation (CLDF) also recommend UDCA for all patients with PBC [17]. However, they recommend adding OCA when ALP > 1.67× ULN, there is a lack of normalisation of bilirubin, being defined as high risk according to UK-PBC/GLOBE PBC predictive models, or evidence of fibrosis progression. The panel recommends that all patients should be considered on a case-by-case basis. Additionally, ACG and CLDF feel that an assessment of the efficacy of UDCA can be made between 6 and 12 months post initiation of UDCA.

### 2.1. Ursodeoxycholic Acid

The first-line therapy for PBC is the physiological BA, UDCA, which is used also for the dissolution of gallstones and the treatment of bile reflux gastritis (Figure 1) [15,33]. It has three proposed mechanisms of action: first, conjugates of UDCA counteract the cytotoxic effects of hydrophobic bile acids on cholangiocytes; second, UDCA stimulates biliary secretion of BAs to maintain healthy bile flow; and third, the inhibition of hepatocyte apoptosis [34,35,36].

Common side effects of UDCA treatment include weight gain, alopecia, gastrointestinal discomfort including diarrhoea, pale stools, and dyspepsia [33]. UDCA is safe for use in breast feeding and pregnancy as there have been no documented adverse events [16].

In 1994, a preliminary double-blinded placebo-controlled RCT (*n* = 129) testing the effectiveness of UDCA on PBC outcomes over 4 years was terminated at 2 years as clinical complications were three times more common in the control group [37]. A significantly reduced incidence rate of LT or death was observed in the UDCA group (*p* = 0.005). This study provided preliminary evidence of the effectiveness of UDCA in PBC by delaying disease progression.

In a later 1994 full-scale double-blinded, placebo-controlled RCT, Heathcote et al. (*n* = 220) found that, within the first 3 months, UDCA therapy significantly reduced serum bilirubin compared to the placebo (*p* < 0.001) [38]. These findings were consistent with another smaller multi-centre RCT by Lindor et al. conducted in 1994 (*n* = 180) (*p* = 0.006) [23]. High serum bilirubin levels are associated with poor prognosis; thus, lowering serum bilirubin may improve clinical outcomes [39]. However, UDCA’s beneficial biochemical profile was not accompanied by histological or symptomatic improvement [38]. A systematic review and meta-analysis may help understand whether UDCA-induced modifications of these markers are associated with a reduction in LT.

In a double-blinded placebo-controlled RCT by Corpechot et al. in 2000 (*n* = 103) to assess the effect of UDCA therapy on histological progression towards fibrosis, a 5-fold lower progression rate from early-stage disease to extensive fibrosis was found in the intervention group at 8 years follow-up (*p* < 0.02) [40]. At completion, 61% of intervention patients remained in the early stage of the disease compared to 13% taking placebo. However, the reduced progression was not accompanied by increased regression in fibrosis. A similar RCT by Poupon et al. in 2003 (*n* = 367) found no overall improvement in histopathological stage with UDCA therapy [41].

These data suggest that implementation of UDCA at the initial stages of PBC may partially improve patients’ fibrosis profile.

Previous studies evaluating UDCA response have often precluded men and young patients from study populations. Multivariate analysis from a cross-sectional study using the Paris criteria by Carbone et al., 2013, (*n* = 2353) showed that men were less likely to respond to UDCA than women (*p* < 0.05) (Table 1) [6]. Increasing age was found to be associated with a better response to UDCA [6]. The large cohort including patients from all hospitals in the UK suggests it was representative of the UK population.

In order to investigate the optimum UDCA dosage, in 1999, Angulo et al. conducted a double-blinded RCT (*n* = 155) to compare the effects of a low (5–7 mg/kg/day), standard (13–15 mg/kg/day), or high (23–25 mg/kg/day) dose of UDCA on liver biochemistry, Mayo score changes, and patient side effect profile [42]. Improvements in ALP (*p* = 0.0001) and Mayo score (*p* = 0.002) were greater in patients administered high and standard doses compared to those administered a low dose. There was no difference between the high- and standard-dose groups. Side effects were more common in the low-dose group indicating they were not dose-related. The most cost-effective dose of UDCA was established at 13–15 mg/kg/day, in concordance with Poupon et al., 1994 [37].

In contrast, a randomised open-label pilot trial (*n* = 80) by Xiang et al., 2021, investigated if UDCA-refractory patients benefitted from increased dosage, from 13 to 15 mg/kg/day to 18–22 mg/kg/day, before initiation of second-line therapy with response defined by the Paris-II criteria (Table 1) [42]. At 6 months, 59.4% of patients in the high-dose group exhibited a complete response compared to 36.1% in standard-dose group. However, the scope of this study was limited by the lack of histological outcomes, leading to an incomplete picture.

Although UDCA is limited by its poor symptom management profile, it is a valid first-line therapy for PBC as it provides significant improvement in key prognostic factors (such as serum ALP) and overall survival with few long-term side effects. Its poor histopathological profile is not in concordance with its other effects.

### 2.2. Obeticholic Acid

OCA is a second-line therapy for the treatment of PBC indicated for 30–40% of patients that do not tolerate or respond to UDCA treatment [6,15,43]. This is defined by the Toronto criteria (Table 1) [15,44]. It is administered at a dose of 5 mg/day and titrated up to 10 mg/day, if well tolerated after 6 months [15].

OCA is derived from the primary human BA chenodeoxycholic acid [45]. It agonises the farnesoid X receptor to reduce bile acid synthesis, liver inflammation, and fibrosis [45,46]. The consequent reduced hepatic bile acid accumulation prevents bile acid toxicity, leading to less cholestatic injury (Figure 1) [47,48].

In 2015, Hirschfield et al. published a proof-of-concept phase 2, double-blinded RCT investigating the efficacy of OCA in 165 PBC patients that were refractory to UDCA therapy [24]. Patients were randomised to receive placebo or were administered OCA doses of 10 mg, 25 mg, or 50 mg while UDCA therapy was continued. All intervention groups observed reduced serum ALP level compared to placebo 3 months later (*p* < 0.0001). Sixty-nine percent of patients receiving an intervention reported at least a 20% decrease in ALP levels in a study powered to detect a mean change greater than 10%.

Another strength of this study is that over 90% of patients are female and Caucasian. This reflects the epidemiological data on PBC patient demographics in clinical practise [49,50]. However, it is not known whether these findings are also valid for other ethnicities and in men.

A 12-month open-label extension of this study reported continued biochemical improvement (*n* = 78). Interestingly, as dosage was increased beyond 10 mg, serum ALP reduction did not improve further (*p* > 0.05) while the incidence of dose-related pruritis became significant (*p* < 0.0003 for 25 mg dose) [24]. Therefore, a guideline dose of 10 mg was established. A 10% dose-related pruritus dropout rate was observed. Therefore, although OCA is efficacious for PBC disease management, its symptomatic management is unimpressive.

Since this open-label extension was underpowered, further long-term investigation of OCA efficacy was justified. Additionally, the primary endpoint of Hirschfield et al. focused on biochemical changes. Disease features such as histopathological change and adverse event rate needed further scrutiny.

The double-blinded, 12-month, phase 3 randomised, “Placebo-Controlled Trial of Obeticholic Acid in Primary Biliary Cholangitis” (POISE) trial assessed the longer-term efficacy and safety of OCA (*n* = 217) according to the Toronto criteria (Table 1) [44,51]. Participants were divided into placebo, OCA 5 mg/day titrated to 10 mg/day after 6 months, and OCA 10 mg/day. The endpoint was reached more often in the intervention groups (*p* < 0.001). This study had sufficient statistical power [51]. Additionally, the Toronto criteria as endpoints was clinically relevant and substantiated the findings of Hirschfield et al. [24]. The POISE trial highlighted a dose-related statistically significant increase in pruritus incidence until 6 months, though, at 12 months, this significance was lost [51]. Therefore, the importance of pruritus as an adverse event requires further investigation.

The interim report at 3 years for the 5-year open-label extension of the POISE trial showed an increase in pruritus incidence from 56% after 1 year to 77% [52]. It was deemed unethical to retain a placebo branch, so no control group comparison is unavailable. After 3 years, trial adherence remained high with an 8% pruritus-related withdrawal rate. These findings alleviated previous concerns regarding adherence. To provide further clarity on adherence and pruritus as an adverse event, the “Phase-IV Study of Obetichicholic Acid Evaluating Clinical Outcomes in Primary Biliary Cholangitis” (COBALT) trial, a long term, phase 4, 10-year trial, is underway (NCT0230811).

An extension of the POISE trial comparing its participants to real-world patients with UDCA-refractory PBC from United Kingdom Primary Biliary Cholangitis Registry showed a reduced progression to liver transplant or death over a 6-year period, reinforcing previous evidence for its efficacy as the second-line agent of choice for PBC treatment.

The COBALT will also investigate the effect of OCA on cardiovascular health. OCA administration causes a drop in high-density lipoprotein although long-term data are unavailable [53]. The clinical significance of an unfavourable OCA cardiovascular profile means that the COBALT trial’s results are keenly awaited [54,55].

Bowlus. C et al. compared liver biopsy data taken at enrolment and after 3 years of OCA treatment and found an improvement or stabilisation of fibrosis [56]. A potential drawback of previous PBC trials is the use of biochemical markers rather than liver biopsy as a primary endpoint [24,51,57]. However, a meta-analysis found that ALP and bilirubin levels can be used to predict PBC outcomes and supported their use as therapy trial endpoints [58]. Although a larger study with a larger sample size evaluating histological data would be useful in further establishing the efficacy of OCA, the current methods and endpoints are sufficient to provide convincing results. OCA remains a validated second-line treatment in disease-modifying management, but, like UDCA, it falls short on symptom management. Further alternate therapies should be considered due the high cost of OCA incurred by the NHS [33].

### 2.3. Alternate Therapies

Several alternative second-line therapies have shown promise in clinical trials. These include antilipemic agents (bezafibrate (BF), fenofibrate), triple therapy (UCDA, steroids, and immunosuppressant), budesonide, and newer agents such as elafibranor and seladelpar.

#### 2.3.1. Bezafibrate

BF treats PBC through its action as a bile acid-sequestrant [59]. The potential therapeutic effect of BF with UDCA in patients with UDCA-refractory PBC has been known for two decades, but it remains off-label. Kanda T et al. reported a RCT of BF (400 mg/day) vs. placebo in 22 patients with elevated serum ALP despite the continuation of UDCA therapy [60]. The intervention group mean serum ALP reduction of 390 IU/L was significant (*p* < 0.01) compared to a 10 IU/L increase in the control.

These preliminary findings were replicated in an RCT with long-term follow-up. In 2015, Hosunuma et al. conducted an open-label RCT of Japanese patients (*n* = 27) with a serum ALP level above 350 IU/L after 24 weeks of continued UDCA administration, by comparing BF 400 mg/day with placebo [61]. They found that, at 9 years, the serum ALP was significantly lower in the intervention group (290 IU/L vs. 462 IU/L, *p* < 0.05). However, there was no change in overall survival. The intervention group had a significantly higher serum creatinine (0.94 mg/dL vs. 0.54 mg/dL, *p* < 0.05), possibly secondary to muscular hyperproduction [15]. Although it is promising that BF is effective long-term, this study was underpowered.

In 2018, the bezafibrate in combination with UDCA in primary biliary cholangitis (BEZURSO) trial, a French double-blinded, placebo-controlled, phase 3 RCT of 100 patients refractory to UDCA, which was continued, investigated BF compared to placebo over 24 months [62]. The primary outcome was the normalisation of the biomarkers: total bilirubin, ALP, AST, ALT, and albumin alongside a normal prothrombin index. This was met in 31% of the intervention-group and 0% of the placebo-group patients (*p* < 0.001). Despite significant improvements in biomarkers, there were no comparable changes in the histological (*n* = 28) fibrosis stage or activity grade. Adverse events were more frequently reported in the controls, suggesting that BF plus UDCA is well tolerated

BF is not yet recommended by the British Society of Gastroenterology guidelines [15]. The guidance used the findings of a 2015 meta-analysis, which found that BF was not associated with any significant improvement in overall survival [63]. The guidance had concerns regarding the lack of data on adverse events and BF’s failure to control liver fibrosis markers (except ALP). The BEZURSO findings reduced these concerns while acknowledging that it does not improve mortality, and BF is now available off-label in the UK. It is possible that, as PBC has a low incidence rate and a relatively long overall survival, this may result in not detecting any changes in mortality rates unless studied in a very large sample size over several decades [18].

It is better to evaluate clinical outcomes such as symptomatic control. In 2021, Elsemieke et al. conducted a double-blinded, placebo-controlled RCT of 74 patients with PBC and primary/secondary sclerosing cholangitis in the Netherlands [64]. By comparing 400 mg/day BF to placebo for 21 days, a 45% decrease in patient-reported pruritis was found in the BF group compared to the placebo group. Though the patient cohort was mixed, a post hoc analysis showed that, in patients with PBC, there was 55% decrease in pruritis. Furthermore, a positive histological change was recorded in a 2020 longitudinal study of 59 patients refractory to UDCA that were receiving 400 mg/day BF [65]. Overall, there is new evidence to suggest that BF has a place as an on-label therapy for PBC, though its association with elevated serum creatinine needs further investigation.

A 2021 retrospective observational study comparing fibrates to OCA found a consistent significantly lower serum ALP decrease in the fibrate group over the 1-year study period (*n* = 346) [66]. Key PBC side effects such as pruritis were less common in the fibrate group. This study indicates bezafibrate could perform favourably compared to the established second-line option.

#### 2.3.2. Fenofibrate

Fenofibrate is also a bile acid-sequestrant. A recent RCT explored the use of fenofibrate as an adjunct first-line therapy alongside UDCA in treatment-naïve patients against UDCA alone [67]. Innovations exploring the modifications of the established first-line therapy are valuable as they will be pertinent to all patients with PBC and not only those refractory to UDCA. Although fenofibrate administration demonstrated a better biochemical response over a 12-month period, this was not replicated for markers of fibrosis or biochemical markers other than ALP. Additionally, the intervention group had a greater number of individuals with elevated liver enzymes at baseline.

#### 2.3.3. Budesonide

As an immunosuppressive steroid, budesonide, could play a role in PBC therapy through the control of autoimmune hepatocellular inflammation. In 1999, Leushner et al. conducted a prospective, placebo-controlled, double-blinded RCT of 39 patients in Germany which found that the budesonide (9 mg/day) arm had a consistent significantly greater decrease in serum ALP compared to the placebo arm (*p* < 0.05) over 24 months [68]. Liver histology evaluated with the Ludwig criteria improved in the intervention group compared to that in the control (*p* < 0.001). Encouragingly, there were no significant changes in bone mineral density (BMD) between groups, which both PBC and steroids are associated with [69]. This study provided strong preliminary evidence for the efficacy of budesonide.

However, in 2006, Rautiainen et al. conducted an open-label RCT of 77 patients with stage 1–3 PBC in Sweden comparing budesonide (6 mg) to UDCA therapy (15 mg/kg) and found no significant serum ALP improvement compared to UDCA (*p* = 0.71) [70]. A low dosage of 6 mg budesonide was used, potentially reducing response. This study suggests budesonide may not be an effective second-line therapy.

In 2021, Hirschfield et al. conducted a double-blinded, placebo-controlled RCT of 62 patients with UDCA-refractory PBC in the UK to compare budesonide (9 mg/day) against a placebo by comparing liver biopsies before and 36 months after intervention. UDCA was continued [31]. The primary outcome of liver histology improvement according to the Ludwig criteria was not compared with the placebo group (*p* = 0.36). However, significantly greater reductions in serum ALP were seen in the intervention group. This trial showed mix results for the efficacy of budesonide.

The evidence for the use of budesonide as a second-line therapy for PBC is limited as the data on serum ALP histological improvement is mixed and there are not enough data to assess symptomatic control. Further feasibility trials to clarify its efficacy are needed before large-scale studies can be considered. Budesonide’s future in PBC treatment is potentially as a component of PBC triple therapy.

#### 2.3.4. Triple Therapy

As PBC is an autoimmune disorder, treatment with immunosuppressives may be of therapeutic value. In 1998, Wolfhagen et al. published a double-blinded placebo-controlled RCT in which 50 patients with UDCA-refractory PBC from the Netherlands additionally received prednisolone (30 mg/day tapered to 10 mg at 8 weeks) and azathioprine (50 mg/day) or placebo over 1 year [71]. The intervention reported a reduction in serum ALP (*p* = 0.002) and pruritus (*p* = 0.05) compared to control over the trial period (*p* = 0.002). A Ludwig’s criteria-derived histological score was better in the intervention group (*p* < 0.001). However, liver biomarkers returned to pre-treatment levels 3 months after treatment termination. Long-term steroid and immunosuppressive therapy are suboptimal and less viable if it must be lifelong. However, the results show convincing evidence for the efficacy of triple therapy.

A 2010 cohort study investigated relevant biomarkers and biopsy data changes in 15 patients UDCA-refractory PBC over 3 years after the additional administration of budesonide (6 mg/day) and mycophenolate mofetil (MMF) (1.5 g/day) (*n* = 38) [72]. A significant reduction in ALP was observed (*p* = 0.0001), alongside pruritus (*p* = 0.05) and histological activity according to the METAVIR scoring system (*p* = 0.002). Despite convincing results, this study’s scope is limited by a small cohort without a placebo arm. However, it provides good pilot evidence for further research.

#### 2.3.5. Novel Agents

Key novel agents such as elafibranor and seladelpar have been shown to be efficacious in PBC treatment, which significantly reduced serum ALP in patients with UDCA-refractory PBC compared to placebo [73,74].

Seladelpar is a novel peroxisome proliferator-activated receptor (PPAR) agonist being explored for the treatment of PBC. PPAR-delta is a hormone receptor that modulates lipid metabolism. PPAR-delta is a broadly expressed, fatty acid-activated transcription factor involved in fatty acid metabolism and inflammation. Seladelpar has been shown to be safe in phase 2 trials, with the added benefit of improving PBC-associated pruritis [75]. The phase 3 ENHANCE and RESPONSE RCTs were of comparable size (*n* = 265 and *n* = 183, respectively) and concordantly showed improvement in symptoms and liver enzyme profile compared to UDCA continuation or placebo in patients with UDCA-refractory PBC [76,77]. Seladelpar’s favourable side effect profile, while improving histological outcomes, is novel; however, since the second-line efficacy of OCA is well established, further trials comparing second-line agents, rather than with placebos, are required.

Elafibranor competes with seladelpar as an alternative second-line PBC therapy. The ELATIVE RCT of 161 patients showed it was more effective than placebo in achieving ALP normalisation, yet, unlike seladelpar, it had no effect on PBC-related pruritic [78]. There is a smaller evidence base for elafibranor than seladelpar.

Linierxibiat and setaxinib are novel therapies shown to be safe, but lacking phase 3 trial evidence for further consideration [79,80]. Linerixibat a minimally absorbed oral small-molecule ileal bile acid transporter (IBAT) inhibitor has been reported to attenuate cholestatic pruritus associated with PBC. Setanaxib is a NOX1/4 inhibitor that has shown anti-fibrotic effects in in vitro and animal studies. However, it is currently being investigated for its efficacy and safety in patients with PBC.

## 3. Discussion

PBC management involves disease-modifying pharmacotherapies. Ursodeoxycholic acid (UDCA) and obeticholic acid (OCA) are the only two drugs recommended by international guidelines and are the established first- and second-line PBC therapies, respectively (Figure 1) [15,16,17]. Despite a poor symptomatic management profile, there is a strong evidence base for UDCA efficacy and safety [81].

OCA is effective in UDCA non-responders as shown by the large POISE and COBALT trials [15,16,17,42]. OCA is effective in controlling serum ALP alongside other prognostic indicators but is contraindicated in patients with cirrhosis. It is expensive and has a poor side effect profile, justifying the need for further second-line options [33].

Fibrates, in particular, BF, remains off-label but have a favourable side effect and serum ALP profile compared to OCA. Its efficacy has a good evidence base, but it has not been compared to OCA directly yet [10,15].

The above disease-modifying therapies provide no effective symptom management [23,24]. Cholestatic pruritus can be managed with cholestyramine or rifampicin. Fatigue management is complex as it could also be secondary to other autoimmune comorbidities such as hypothyroidism, which must be excluded [10,15,25,26,27,28,29]. Antilipemic agents and steroids may play a future role in PBC management [30,31].

There is a therapeutic gap in PBC for individuals who do not respond to UDCA and cannot tolerate OCA. Seladelpar and elafibranor (PPAR agonists) are options that require longitudinal clinical trials to establish long-term safety and efficacy. Sequential systematic reviews and meta-analyses of these agents to aggregate their efficacy data may justify the direct comparison to OCA and UDCA as adjuncts or alone therapies. A recent network meta-analysis predicted that elafibranor and USDA combination therapy might be the most effective second-line option but was unable to directly compare therapeutic agents [82].

There may be some possible limitations in this literature review as it exclusively focuses on PBC. For example, this review may be confounded by coexisting diseases such as autoimmune hepatitis overlap [83]. Additionally, treatments for symptomatic management of PBC were not discussed.

Looking forward, ongoing clinical trials, ideally directly comparing candidate second-line therapies, will provide more options for disease-modifying therapies and symptomatic control. Future therapeutic options will help to prevent disease progression and improve the quality of life in patients with PBC. 

## Figures and Tables

**Table 1:** Different criteria for analysis of response to UDCA treatment.

Criterion	Definition of the Biochemical Response
Paris-I	ALP ≤ 3× ULN, AST ≤ 2× ULN, and normal bilirubin after 1 year of UDCA
Paris-II	ALP and AST ≤ 1.5× ULN with normal bilirubin after 1 year of UDCA
Barcelona	ALP decrease > 40% from baseline or to normal after 1 year UDCA
Toronto	ALP ≤ 1.67× ULN or ALP ≤ 1.76× after 2 years of UDCA

ALP, alkaline phosphatase; UDCA, ursodeoxycholic acid; AST, aspartate transaminase; and ULN, upper limit of normal.

**Table 2:** Search terms used to find relevant literature in Pubmed, Ovid Medline, and Web of Science.

UDCA	(“Ursodeoxycholic acid” OR “UDCA” OR “ursodiol”) AND (“Primary Biliary” OR “Primary Biliary Cirrhosis” OR “Primary Biliary Cholangitis” OR PBC”)
OCA	(“Obetacholic acid” OR “OCA” OR “Ocaliva” OR “6alpha-ethyl-chenodeoxyxholic acid” OR “6-ECDCA”) AND (“Primary Biliary” OR “Primary Biliary Cirrhosis” OR “Primary Biliary Cholangitis” OR PBC”)
Bezafibrate	(“Bezafibrate” OR “Fenofibrate”) AND (“Primary Biliary” OR “Primary Biliary Cirrhosis” OR “Primary Biliary Cholangitis” OR PBC”)
Budesonide	(“Budesonide”) AND (“Primary Biliary” OR “Primary Biliary Cirrhosis” OR “Primary Biliary Cholangitis” OR PBC”)
Triple Therapy	(“Triple Therapy” OR (“Azathioprine” OR “Prednisolone” AND (“UDCA” OR “Ursodeoxycholic acid” OR Ursodiol”)) OR (“Mycophenolate Mofetil” OR “Budesonide” AND (“UDCA” OR “Ursodeoxycholic acid” OR Ursodiol”))) AND (“Primary Biliary” OR “Primary Biliary Cirrhosis” OR “Primary Biliary Cholangitis” OR PBC”)
NOT terms	“Primary Sclerosing” AND “PSC”

**Table 3:** Current and emerging medical therapies in primary biliary cholangitis.

**Current Licenced Therapies**	
Ursodeoxycholic acid	The first-line therapy for PBC has three proposed mechanisms of action: firstly, conjugates of UDCA; secondly, UDCA stimulates the biliary secretion of BAs, and lastly, the inhibition of hepatocyte apoptosis.
2. Obeticholic acid	The second-line therapy for PBC treatment is derived from the primary human BA chenodeoxycholic acid, and it agonises the farnesoid X receptor.
**Alternate Therapies**	
Bezafibrate	It treats PBC through its action as a bile acid-sequestrant. Although it is not yet recommended by the British Society of Gastroenterology guidelines, clinical data suggest it could perform well as a second-line option.
2. Fenofibrate	It treats PBC by acting as a bile acid-sequestrant. It is currently being explored as an adjunct first-line therapy alongside UDCA in treating naive patients against UDCA alone.
3. Budesonide	As an immunosuppressive steroid, budesonide could have a role in PBC therapy through control of autoimmune hepatocellular inflammation. However, the evidence for the use of budesonide as a second-line therapy for PBC is limited. Budesonide’s future in PBC treatment is potentially as a component of PBC triple therapy.
4. Triple therapy	As PBC is an autoimmune disorder, the addition of immunosuppressive therapies to UCDA could be of a therapeutic value. However, further research is needed in this area.
**Emerging novel therapies**	
Elafibranor and Seladelpar	Seladelpar and elafibranor are novel peroxisome proliferator-activated receptor (PPAR) agonist being explored for the treatment of PBC. PPAR-delta is a hormone receptor that modulates lipid metabolism.
Linierxibiat	Linerixibat, a minimally absorbed, oral, small-molecule ileal bile acid transporter (IBAT) inhibitor has been reported to attenuate cholestatic pruritus associated with PBC.
Setaxinib	Setanaxib is a NOX1/4 inhibitor that has shown anti-fibrotic effects in in vitro and animal studies. However, it is currently being investigated for its efficacy and safety in patients with PBC.

**Table 4:** A summary of the current international guidelines regarding the management of PBC from EASL, BSG, and ACG.

Clinical Guidelines	EASL	BSG	ACG
First-Line management	UDCA 13–15 mg/kg/day	UDCA 13–15 mg/kg/day	UDCA 13–15 mg/kg/day
Criteria to consider before starting second-line therapy	Biochemical response post 1 year of UDCA treatment: ALP > 1.67× ULN and/or bilirubin elevated < 2× ULN	Biochemical response post 1 year of UDCA treatment: ALP > 1.67× ULN and/or bilirubin < 2× ULN	Biochemical response post 6–12 months of UDCA treatment: persistently raised ALP after 12 months; lack of normalisation of bilirubin with UDCA; patient defined as high risk according to UK-PBC/GLOBE PBC; proof of fibrosis progression.
Second-line management	OCA Starting with 5 mg dose once daily and titrated to 10 mg once daily, if tolerated	OCA Initial dose of 5 mg/day, titrating to 10 mg/day at 6 months, if tolerated	OCA Starting with 5–10 mg once daily (in patients with Child–Pugh Class B/C, OCA 5 mg once weekly to 10 mg twice weekly)
Alternative therapies	Unlicensed therapies are not recommended by the EASL	Unlicensed therapies are not recommended by the BSG	If the above are not successful, patients should be considered for clinical trials.

EASL, European Association for the Study of the Liver; BSG, British Society of Gastroenterology; ACG, American college of gastroenterology; UDCA, Ursodeoxycholic acid; ALP, Alkaline phosphatase; ULN, upper limit of normal; OCA, Obetacholic Acid

## Data Availability

No new data were generated for this study.
